# Red ear syndrome precipitated by a dietary trigger: a case report

**DOI:** 10.1186/1752-1947-8-338

**Published:** 2014-10-10

**Authors:** Chung Chi Chan, Susmita Ghosh

**Affiliations:** 1 Department of Audiovestibular Medicine, St Ann’s Hospital, St Ann’s Road, London N15 3TH, UK; 2 Department of Adult Audiovestibular Medicine, Royal National Throat, Nose and Ear Hospital, 330 Grays Inn Road, London WC1X 8DA, UK; 3 Department of Audiovestibular Medicine, Platt Bridge Health Centre, Rivington Drive, Bickershaw, Wigan WN2 5NG, UK

**Keywords:** Dietary trigger, Erythema, Lifestyle modifications, Migraine, Red ear syndrome

## Abstract

**Introduction:**

Red ear syndrome is a rare condition characterized by episodic attacks of erythema of the ear accompanied by burning ear pain. Symptoms are brought on by touch, exertion, heat or cold, stress, neck movements and washing or brushing of hair. Diagnosis and treatment of this condition are challenging. The case we report here involves a woman whose symptoms were brought on by a dietary trigger: orange juice as well as stress, causing significant physical and psychological morbidity. Avoidance of triggers resulted in symptomatic improvement.

**Case presentation:**

A 22-year-old Caucasian woman who was a student presented twice to our department with evolving symptoms, the first time with hyperacusis (abnormal sound sensitivity arising from within the auditory system to sounds of moderate volume), intermittent right tinnitus and subjective hearing difficulties. She presented five years later with highly distressing episodes of erythematous ears, which were associated with burning pain around the ear and temporal areas, and intolerance to noise. After keeping a symptom diary, she identified orange juice and stress as triggers of her symptoms. No local head and neck pathology was present. Investigations and imaging were negative. Avoidance of triggers led to great symptomatic improvement. To the best of our knowledge, dietary triggers have not previously been reported as a trigger for this syndrome. This case shows a direct temporal link to a dietary trigger and supports a primary pathogenesis. Recognition and management of primary headache disorder and simple dietary and lifestyle changes brought about symptomatic relief.

**Conclusion:**

Red ear syndrome is a little-known clinical syndrome of unknown etiology and management. To the best of our knowledge, our present case report is the first to describe primary red ear syndrome triggered by orange juice. Clinical benefit derived from avoidance of this trigger, which is already known to precipitate migraines, gives some insight into the pathogenesis of red ear syndrome.

## Introduction

Red ear syndrome (RES) is a rare condition characterized by episodic erythema of the ear accompanied by burning sensation or otalgia. One or, less commonly, both ears may be affected, and erythema may extend beyond the ear to the face. Symptoms may be spontaneous or triggered by touch, exertion, heat or cold, stress, neck movements, sneezing, coughing, chewing and/or brushing of hair [1]. Recognition of this condition is important but difficult because of its rarity.

## Case presentation

A 22-year-old Caucasian woman who was a student presented to our neuro-otology clinic on two separate occasions five years apart. Her initial symptoms were a six-month history of intermittent right-sided tinnitus and bilateral hyperacusis (abnormal sound sensitivity arising from within the auditory system to normal or moderate-level ambient noise which would not trouble other people). She also reported right ear fullness and significant difficulty hearing in background noise when stressed.

**Figure 1:**
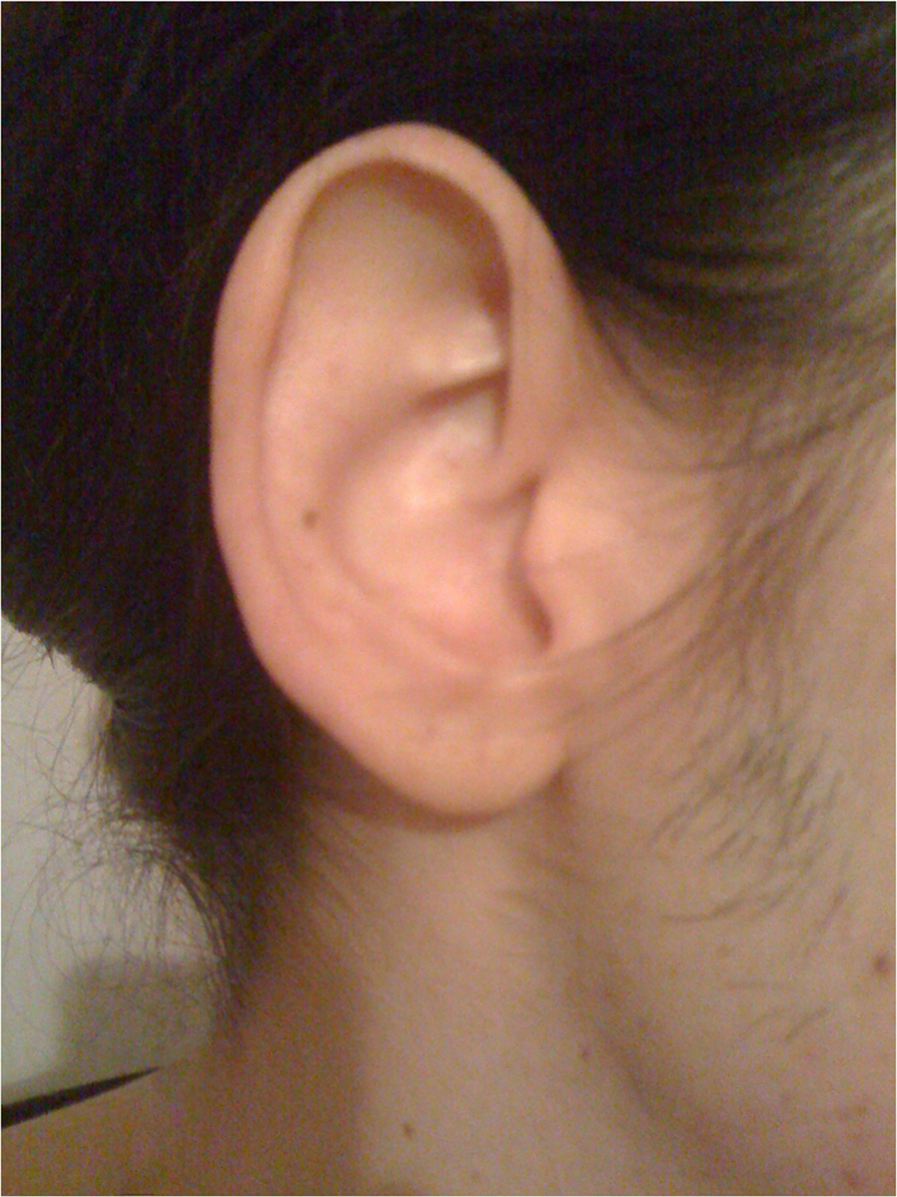
Normal appearance of the right ear of the patient (photograph taken by patient).

**Figure 2:**
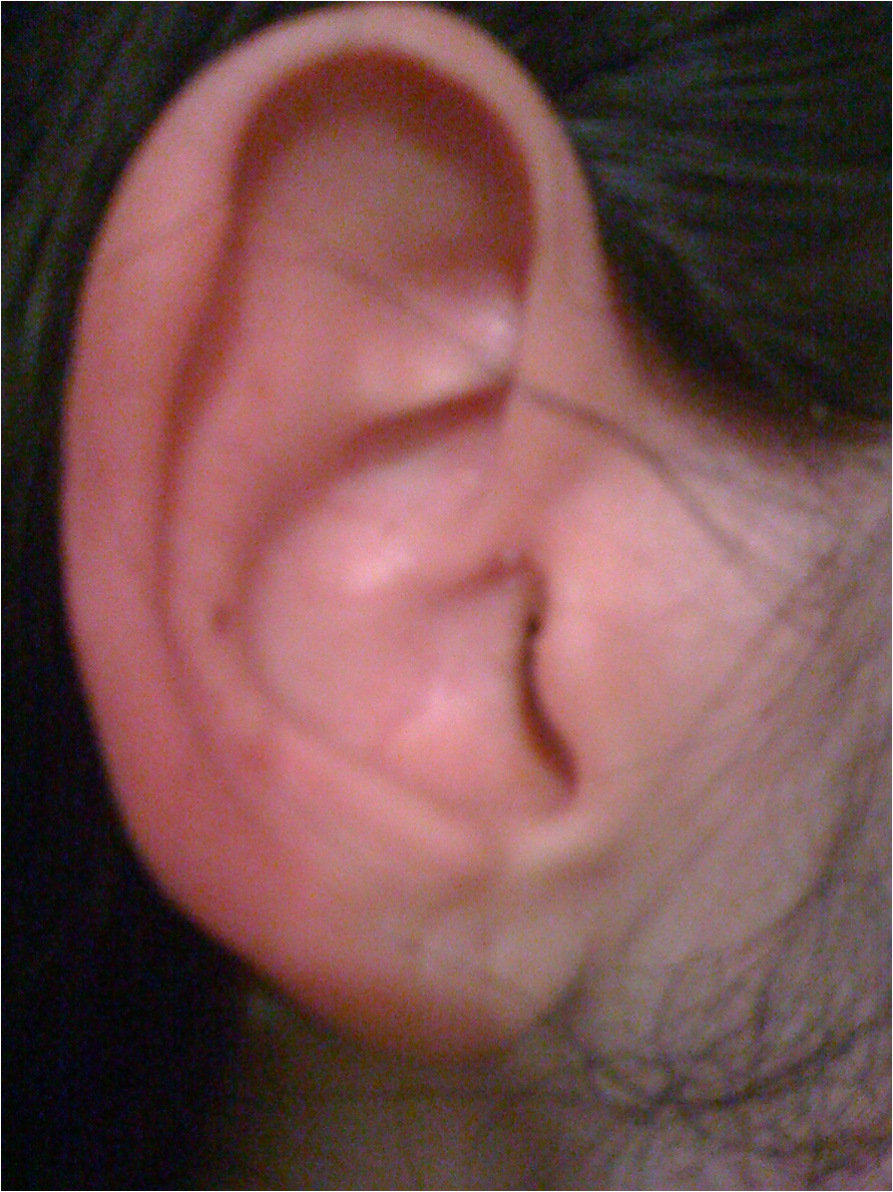
Right ear during the patient’s “red ear” episode (photograph taken by patient).

Otoscopy, a neuro-otological examination, pure-tone audiometry, tympanometry, stapedial reflexes, oto-acoustic emissions (OAEs), auditory brainstem response and speech audiometry results were normal. She had particularly strong transient OAE responses and spontaneous OAE activity bilaterally. This was consistent with increased cochlear gain, suggestive of reduced efficacy of inhibitory feedback in the auditory system. Several sessions of auditory rehabilitation were carried out with a hearing therapist, involving counseling, communication tactics, tinnitus and hyperacusis retraining, advice regarding ear-level noise generators to enable desensitization, relaxation techniques and stress management. Her symptoms had greatly improved at review nine months later, and she was discharged.She was referred again to our clinic by her general practitioner five years later. Her primary complaint was recurrent one-hour episodes of painful cutaneous erythema of the right external ear (Figures 1 and 2) that was associated with severe right temporal pain radiating down to the mastoid area with transient subjectively reduced hearing, right conjunctival injection, intolerance to noise and light, which was exacerbated during these episodes; the latter symptom was suggestive of involvement of pathways outside the auditory pathway.

These symptoms caused our patient considerable distress, resulting in weekly attendance for three months at her general practitioner’s clinic, in addition to presentation at the local emergency departments and to ear, nose and throat clinics, prior to referral to our department. During the previous three months, she had also experienced continuous headaches and fatigue with occasional light-headedness during episodes of erythematous ear. She reported no nausea, visual field symptoms, tinnitus or vertigo.

Differential diagnoses of dermatological, temporomandibular joint, dental, pharyngeal and cervical problems were excluded on the basis of a head and neck examination. On inspection, there was no evidence of erythema or of infection in the ear or mastoid area. The otoscopy findings were normal. A neuro-otological examination was unremarkable, including extra-ocular eye movements, cranial nerves, cerebellar function and clinic room balance tests. Pure-tone audiometry and tympanometry showed normal hearing and middle-ear function. Magnetic resonance imaging of the brain was normal. Routine blood tests were negative. She was diagnosed with RES associated with hyperacusis.

She was reassured that she had no major structural pathology. There were some migrainous features in her medical history. Management of her migraine included starting behavioral modifications, such as reducing caffeine intake, stress reduction, optimizing fluid intake, improving sleep pattern, relaxation techniques and starting exercise. She was advised to keep a symptom diary to identify further triggers. She was offered migraine prophylaxis but declined it.

On review after four months of supportive measures, she was feeling much better, with complete resolution of her headaches and much reduced frequency of episodes of erythematous ear. She reported absence of pain in the ear and less sensation of swelling. Triggers for red ear episodes identified from her symptom diary included stress and, surprisingly, orange juice. Her symptoms were managed successfully without medication for four years.

## Discussion

RES is a clinical diagnosis for which there is no specific diagnostic test. One hundred cases have been reported in the literature so far, with an estimated male-to-female ratio of 1:1.25 and a median age at onset of 44 years (with a wide range of ages: 4 to 92 years) [1]. Lance [2] was the first to describe RES in the literature in a 1996 report of 12 patients with recurrent RES. Pain in RES varies from mild to severe [3,4]. Its duration may be seconds [5] or hours [2]. The frequency of episodes may be several times per day, or there may be year-long remission periods [6]. Raieli and colleagues [4] reported that unilateral or bilateral 30- to 60-minute episodes can occur in isolation and be associated with migraine (before, during or after). In 10% of the cases they reported, red ears preceded onset of a painful migraine attack. In their series of 96 children admitted with headache, who ranged from 6 to 18 years of age, 55 (57%) had migraine, and RES was found in 16 migraine cases. RES did not occur in the other headache groups. It was associated with severe pain in 62.5% and neurovegetative symptomatology (nausea, vomiting, phonophobia and/or photophobia) in 50% [4].

Episodes have been reported to occur spontaneously or to be triggered by heat [2]; by entering a hot room [7]; by touch [2,5]; by neck movement [2,5]; by sneezing, coughing, hair-brushing, physical exercise, chewing, and stress [2]; and by exposure to cold and lying on the affected side [8].

There are various views regarding the pathophysiology of this condition. Lance [2] suggested that the syndrome is induced in patients with cervical disorders, predominantly C3 root discharge causing antidromic release of vasodilator peptides (peripheral mechanism). He proposed that the primary mechanism is activation of the trigeminovascular system. He pointed out, and Hirsch [9] reiterated, that parasympathetic vasodilatation is greater in the nose and cheek than in the ear; therefore, red ears must be mediated primarily by inhibition of sympathetic vasoconstriction or activation of sympathetic vasodilatation. Thus, the presence of RES suggests an underlying dysregulation of sympathetic outflow. Purdy [10] noted that, in RES, there is pain in and around the ear associated with autonomic phenomena, including erythema of the ear ipsilateral to the pain. He suggested that the condition be labeled auriculoautonomic cephalalgia or be placed in the trigeminal autonomic cephalgia group. Several authors, including Kumar and colleagues [5], have used brainstem trigeminovascular activation to explain RES associated with migraine. Lambru and colleagues suggested that it is possible that trigeminoautonomic parasympathetic activation occurs with sympathetic deficit. The imbalance between parasympathetic and sympathetic systems thus may facilitate inhibition of sympathetic tone of the ear. Sympathetic dysregulation, not parasympathetic activation as formerly believed, may be the predominant mechanism of RES [1].

Another group [7] has suggested that RES is an auricular form of erythromelalgia with similar burning pain, erythema and increased skin temperature. Erythromelalgia is a condition affecting hands and feet that might be caused by sensory and sympathetic nerve dysfunction.

RES has been associated with various conditions, including upper cervical pathology (arachnoiditis, facet joint spondylosis and cervical root traction), glossopharyngeal and trigeminal neuralgia, temporomandibular joint (TMJ) dysfunction and thalamic syndrome [2]. Associations have been reported with primary headache disorders, including migraine, chronic paroxysmal hemicranias [3], hemicrania continua and the short-lasting unilateral neuralgiform headache with conjunctival injection [11]. Other cases are idiopathic. Donnet and Valade proposed two types of RES: (1) a primary form that occurs in young people and is associated with migraine and (2) a secondary form that occurs in older adults and is associated with cervical disorder or trigeminal autonomic cephalalgia phenomenon [6].

Various treatments for RES have been used with varying success. Among the 12 patients Lance described, one improved with methysergide therapy. One experienced partial symptomatic relief with indometacin, and others with propranolol, application of a cold pack, amitriptyline, or imipramine [2].

Inconsistent results have been reported following treatment with non-steroidal anti-inflammatory drugs [12], topical anesthetics, cooling the ear [7], verapamil and gabapentin [8] and greater auricular nerve blockade with a combination of local anesthetics and steroids. Some authors have reported relief over an eight-week period, and others have noted no benefit [2,13]. Bender [14] suggested that in primary or idiopathic cases, treatment of the coexisting headache disorder with drugs such as propranolol, amitriptyline, imipramine and flunarizine helps to resolve these cases to varying degrees. Secondary forms may be more resistant to treatment [12]. In one review, secondary cases appeared to have a greater response to treatment than idiopathic cases [15]. In secondary cases, such as those associated with TMJ dysfunction, a dental plate was reported to be useful in relieving symptoms [2,9]. In cases associated with chronic paroxysmal hemicrania, indometacin was found to be effective [3,16].

Transient unilateral sensation of aural fullness with tinnitus was described in one of Lance’s original cases [2] and blockage without tinnitus in 2 further cases of chronic paroxysmal hemicrania [3]. Aural fullness was present in our case on the first presentation; additional subjective hearing loss in background noise without evidence of hearing loss on audiometry was possibly due to central auditory pathway changes.

Various triggers of RES have been identified; however, we found no reports of dietary triggers that provoke RES. Alcohol and spicy foods are known to cause bilateral facial flushing. Gustatory flushing is mediated by an autonomic neural reflex involving the trigeminal nerve. The presence of a dietary trigger that causes neurological symptoms suggests a migrainous etiology, as such triggers in migraine are well-known and avoidance is a therapeutic mainstay. The non-concentrate orange juice that our patient consumed is a well-known brand in the United Kingdom. There is a possibility that ethyl butyrate (also known as butyric acid ethyl ester), which is often used in flavoring extracts, could be the culprit rather than the orange juice itself. In our patient, dietary, stressor and lifestyle modifications were sufficient to relieve her physical symptoms and psychological distress. We advocate examining a patient’s lifestyle and encouraging the patient to keep a symptom diary to identify environmental factors that provoke RES. Clinicians should be made aware of the likelihood of migraine pathogenesis in primary RES and the range of management options available (non-pharmacological lifestyle changes as well as prophylaxis).

RES is a little known condition with much variation in individual patients’ symptoms and variable responses to proposed treatments. It may go unrecognized and can cause the patient undue anxiety. Often patients feel ignored without a firm diagnosis or management. Patients may present repeatedly to emergency departments, general practices or various specialist departments (ear, nose and throat; dermatology; neurology; audiovestibular medicine; and/or audiology) before being diagnosed. Raised awareness of this disorder with prompt diagnosis has cost benefits by reducing the number of primary and secondary care presentations, decreasing psychological distress and speeding return to usual daily activities.

## Conclusions

RES is a rare syndrome of diverse pathophysiology which is difficult to treat. To our knowledge, our present report is the first to describe a dietary trigger of RES. Successful management with lifestyle modifications and avoidance of migraine triggers gives insight into the pathogenesis of primary migraine-associated RES.

## Consent

Written informed consent was obtained from the patient for the publication of this report and any accompanying images. A copy of the written consent is available for review by the Editor-in-Chief of this journal.

## Competing interests

The authors declare that they have no competing interests.

## Authors’ contributions

SG reviewed the literature and wrote the initial manuscript drafts. CC managed the patient, reviewed the literature and completed the manuscript. Both authors read and approved the final manuscript.
